# Comparison of the effects of 17β- estradiol treated and untreated mesenchymal stem cells on ameliorating animal model of multiple sclerosis

**DOI:** 10.22038/IJBMS.2018.29438.7115

**Published:** 2018-09

**Authors:** Rahim Heidari barchi nezhad, Fatemeh Asadi, Mohammad Reza Mirzaei, Seyyed Meysam Abtahi Froushani

**Affiliations:** 1 Molecularmedicine research center, Rafsanjan university of medical sciences,Rafsanjan,Iran; 2 Departement of clinical biochemeistry, Rafsanjan university of medical sciences,Rafsanjan,Iran; 3 Department of Microbiology, Faculty of Veterinary Medicine, University of Urmia, Urmia, Iran

**Keywords:** Experimental autoimmune encephalomyelitis, Immunotherapy, Mesenchymal stem cell, Multiple sclerosis, 17β- estradiol

## Abstract

**Objectives:**

The current investigation was undertaken to evaluate the effects of 17β- estradiol (17β-ED) on the potential of the mesenchymal stem cells (MSCs) for modulation of immunity responses in an animal model of multiple sclerosis (MS).

**Materials and Methods:**

After isolation of MSCs, cells were cultured in presence of 100 nM 17β-ED for 24 hr. Modeling of experimental autoimmune encephalomyelitis (EAE) was achieved by using guinea pig spinal cord homogenate, in addition to complete Freund’s adjuvant in male Wistar rats. The processes of cell therapy were started following 12 days post-immunization. This duration allows all animals to develop a disability score. The achieved EAE clinical symptoms were regularly monitored every day until day 36, when all of examined rats were euthanized.

**Results:**

Cell therapy in the EAE rats with 17β-ED-primed MSCs exhibited more desirable consequences, which in turn lead to regression of the cumulative clinical score and neuropathological changes that are more than the therapy with untreated MSCs. The serum measures of myeloperoxidase (MPO), nitric oxide (NO) as well as splenocytes-originated pro-inflammatory interleukin-17 (IL-17) and tumor necrosis factor alpha (TNF-α) were significantly decreased in EAE rats treated by 17β-ED primed-MSCs compared to EAE rats that received untreated MScs.

**Conclusion:**

Combination of 17β-ED and MSCs more effectively improved the signs and symptoms of EAE.

## Introduction

The neurological disorder of multiple sclerosis (MS) is characterized by an inflammatory, demyelinating and disabling illness of the central nervous system (CNS), which mostly affects young adults. In general, the pathogenesis of MS is considered as an immune system abnormality, which leads to multiple neurological disorders manifestation with autoimmune origin (1). Despite the valuable improvements in the pharmacotherapy of MS, the current therapeutic regiments have exhibited low efficacy, accompanied by unwanted side effects (1, 2). Hence, development of novel curative regimens for MS patients seems necessary. In fact, it is highly essential to note that most of medications that are currently confirmed for MS are developed on the basis of investigations on animal experimental autoimmune encephalomyelitis (EAE) models of the disease (3).

Stem cells with the mesenchymal tissues origin, known as MSCs, are described according to their potential ability for self-renewing. These subtypes of stem cells are multipotent progenitor cells that have the ability to differentiate to different mesenchymal lineages, including either cartilage and bone or fat cells (4, 5). The MSCs are generally isolated from the bone marrow; nevertheless, these cells could accommodate in several other tissues, such as umbilical cord blood and adipose (5, 6), dermal (7), as well as peri-endothelial tissues (8). Compelling scientific reports have provided background evidences that MSCs possess a unique immunoregulatory and regenerative potential (9, 10). It has also been suggested that MSC therapy could be potentially considered as a promising immunomodulatory approach for cell therapy of immune-mediated diseases, including MS (11). In this regard, pervious findings demonstrated that MSCs can promote immunomodulation and neuronal survival, and restore axonal myelination in MS and EAE models (12). Nevertheless, inadequate viability of transplanted cells reaching to inflamed or damaged tissues has limited their potential beneficial effects (13).

Beneficial effects of 17 β -estradiol (E2) administration in ameliorating EAE have been reported in some previous studies (14, 15). Indeed, it is clear that pregnancy casus a significant decrease in the clinical symptoms in women with MS (16). Also, a protective benefit of estrogens has been demonstrated in a pilot clinical trial using estradiol administration in MS patients (17). Biological effects of estrogens are promoted by two nuclear estrogen receptors (ER) ERα and ERβ, which are ubiquitously expressed in the cells like MSCs (18). A new report indicated that conditioning of MSCs with E2 can promote migration of cells in cultured MSCs *in vitro* and in a diabetic rat model *in vivo *(13). However, there is a big gap of information regarding possible beneficial effects of treatment of EAE or MS with E2-primed MSCs. Thus, current investigation was aimed to explore the possible impacts of E2-primed MSCs therapy in EAE animal model of MS.

## Material and Methods

Reagents

The anti-rat CD29 (Integrin β chain; Ha2/5; FITC), CD45-FITC, and CD90-PCY5 (Thy-1/Thy-1.1-FITC) were obtained from BD PharMingen. Fetal calf serum (FCS) and Dulbecco’s modified Eagle’s medium (DMEM) were purchased from Gibco/Life Technologies Inc. Cytokines were assayed employing enzyme-linked immunosorbent assay (ELISA) kits, which were procured by Bender MedSystems. The rest of reagents were obtained from Sigma-Aldrich (St. Louis, MO, USA).

**Animals**

The male albino Wistar rats, aged 6-8 weeks were purchased from animal house of Rafsanjan University of Medical Sciences (Rafsanjan, Iran) and allowed 7 days to acclimate before the start of the study. Animals were kept in controlled environmental conditions (25 ℃ and a 12 hrs light/dark cycle). Rats were fed with standard laboratory chow and had free access to water, *ad libitum*. The welfare condition of rats was established in accordance with the guidelines of the National Institute of Health Guide for the Care and use of laboratory animals.

**Induction and treatment of EAE rats**

EAE was induced according to the method described earlier (12). Briefly, the spinal cords were gently dissected out from the anesthetized guinea-pigs. One gram of frozen guinea pig was homogenized by grinding in 1 ml of 0.9% NaCl for 4 min at room temperature. Rats were immunized by a subcutaneous injection (0.4 ml) in the flanks of guinea pigs’ spinal cord homogenate (GPSCH), emulsified in complete Freund’s adjuvant (CFA), which contained 10 mg/ml of *Mycobacterium tuberculosis* H37Ra (1:1 v/v). The immunization processes were completed by twice (in two phases of immediate and further 48 hr) intraperitoneal (IP) injection of 0.1 ml pertussis germ suspension (1×10^10^ / ml).

Cell therapy was established whenever all of the rats exhibited signs and symptoms of EAE. Following EAE induction, rats were randomly allocated into 4 groups: Vehicle-treated EAE rats, MSC-treated EAE rats, E2 primed MSCs-treated EAE rats, and normal control (n = 10 in each). The MSCs or E2 primed MSCs were intraperitoneally injected (2 × 10^6^ cells/rat) to the MSC-treated group at the initiation phase of therapy. The number of cells (2 × 10^6^ cells/rat) was on the basis of previous investigation on the EAE experimental rat models (19-21). Both the vehicle-treated EAE and normal rats received an equal volume of phosphate-buffered saline (PBS). Noticeably, the normal control animals were similarly treated, as the EAE rats without GPSCH. 

To evaluate the degree of neurological disability, all of rats were daily weighed and monitored and the motor disability was further scored as bellow: 0: free of disease, 1: lack of tail tonicity, 2: tail paralysis, 3: hind limb paralysis, 4: hind and forelimb paralysis, 5: moribund or death (22).

**MSC separation and** **immunophenotyping**

As described previously, MSCs were isolated from the flushed bone marrow of bilateral femurs and tibias from anesthetized Wistar rats (23). Isolated cells were centrifuged and were further cultured in 25-cm^2^ tissue culture flasks in DMEM medium 0.3-0.4 × 10^6^ cells/cm^2^ supplemented with 15% heat-inactivated fetal bovine serum (FBS), penicillin (100 U/ml), and streptomycin (100 mg/ml), at 37 °C in a humidified 5% CO2 incubator. Within the third day, all of non-adherent cells were discarded. The culture supernatant medium was changed, twice a week. The passage was performed at subculture 3 using 0.25% trypsin, and 0.02% EDTA. The isolated cells were further utilized for immunophenotyping by flowcytometry as described earlier (24). Afterwards, the isolated cells were marked by the fluorescent-conjugated monoclonal antibodies including the MSCs markers CD29 and CD90, as well as CD45 as the hematopoietic marker. A Dako flowcytometer was employed for immediate determination of cellular fluorescence (Partec, Germany).

**Treatment of MSCs**

At the passage 3, MSCs were used to perform further experiments. MSCs were left untreated (control) or primed with 100 nM 17β-ED for 24 hr. After aspiration of the medium, the cells were gently rinsed three times with PBS and isolated by 0.25% trypsin, containing 0.02% EDTA. 

**Evaluation of myeloperoxidase (MPO) activity** **in the serum**

The activity level of MPO was examined in accordance with a method described earlier (25). Briefly, blood specimens were aspirated from anesthetized rats using cardiac puncture in sodium citrate (0.129 M; pH 6.5; 9:1, v/v). Volume of 10 µl of isolated serum samples were then added to 80 µl of 0.75 mM H_2_O_2_ and 110 µl Tetramethyl Benzidine (TMB solution; 2.9 mM TMB in 14.5% DMSO and 150 mM sodium phosphate buffer at pH 5.4) and the resultants were cultured at 37 °C for 5 min. Again, 50 µl of 2 M H_2_SO_4_ was added to the combination to terminate the reactions, and the absorbance was spectrophotometrically read at 450 nm. The horseradish peroxidase (HRP) at a volume of 10 µl (2.5 and 25 milliunit/ml HRP) was applied as standard. The MPO activity was calculated as the difference between the HRP absorbance standard curve. Values were presented as milliunits per milliliter (mU/ml).

**Determination of serum nitric oxide (NO) level**

The serum NO levels were defined by using the Griess method. Serum samples (50 µl) were added to 50 µl of Griess reagent (0.1% sulphanilamide, 3% phosphoric acid and 0.1% naphthyl ethylenediamine) and were further cultured at room temperature for 10 min at dark. Finally, the absorbance of samples was read at 540 nm on a microplate reader and the NO concentration was calculated in accordance to the standard curve (26).

**Assessment of the splenocytes-derived cytokines profile**

The rat's spleens were aseptically removed. For this purpose, a single-cell suspension of splenocytes was made up in RPMI 1640 medium supplemented with 10% FCS. The contaminated red blood cells (RBC) were discarded by RBC lysis buffer. A cell suspension (2 × 10^6^ cells/ml) was then provided and incubated in 24-well plates. The phytohemagglutinin (PHA) solution (10 ug/ml) was added to the culture and the resultant culture supernatants were isolated after 72 hr. The supernatant levels of interferon-γ (IFN-γ), interleukin-17 (IL-17), tumor necrosis factor alpha (TNF-α), and IL-10 were determined by ELISA, on the basis of the kit manufacturer's guidelines (1). 

**Neuropathology**

The animals were euthanized under deep anesthesia at the day 36 of immunization. Spinal cord specimens (cervical, thoracic, and lumbar parts) were dissected and fixed in 10% buffered formalin and embedded in paraffin. Spinal cord sections (5 mm thick) were stained by hematoxylin and eosin. Nine transverse sections of spinal cord specimens were provided (three from cervical, three from thoracic, and three from lumbar, each chosen from every 15 slides). The provided pathology sections were checked by microscope (Olympus BX40, 200x magnification), and digital images were captured using Axio-Cam (Zeiss, Oberkochen, Germany) and further evaluated by Zeiss software (AxioVision 40 4.7). The percentage of lesion size in each slide was calculated based on the ratio of white mater lesion to the whole white matter area. The mean percentage of lesion size in all of three parts of the spinal cord sections in each group was further reported (12).

**Statistical analysis**

To statistically analyze the collected data, the spss software version 21.00 was applied. We also employed non-parametric statistical methods for analysis of the clinical scores. The ranks among the experimental and the ranks within the experimental groups were analyzed by applying a Kruskal–Wallis test along with the non-parametric statistical method of Mann–Whitney U test with bonferroni adjustment for multiple comparisons. We also used the one-way Analysis of variance (ANOVA) test, and relatively mean comparison were performed by Tukey *HSD hoc* test. All of the obtained data were expressed as mean ± SD, and *P*<0.05 was regarded significant.

## Results

**Figure 1:**
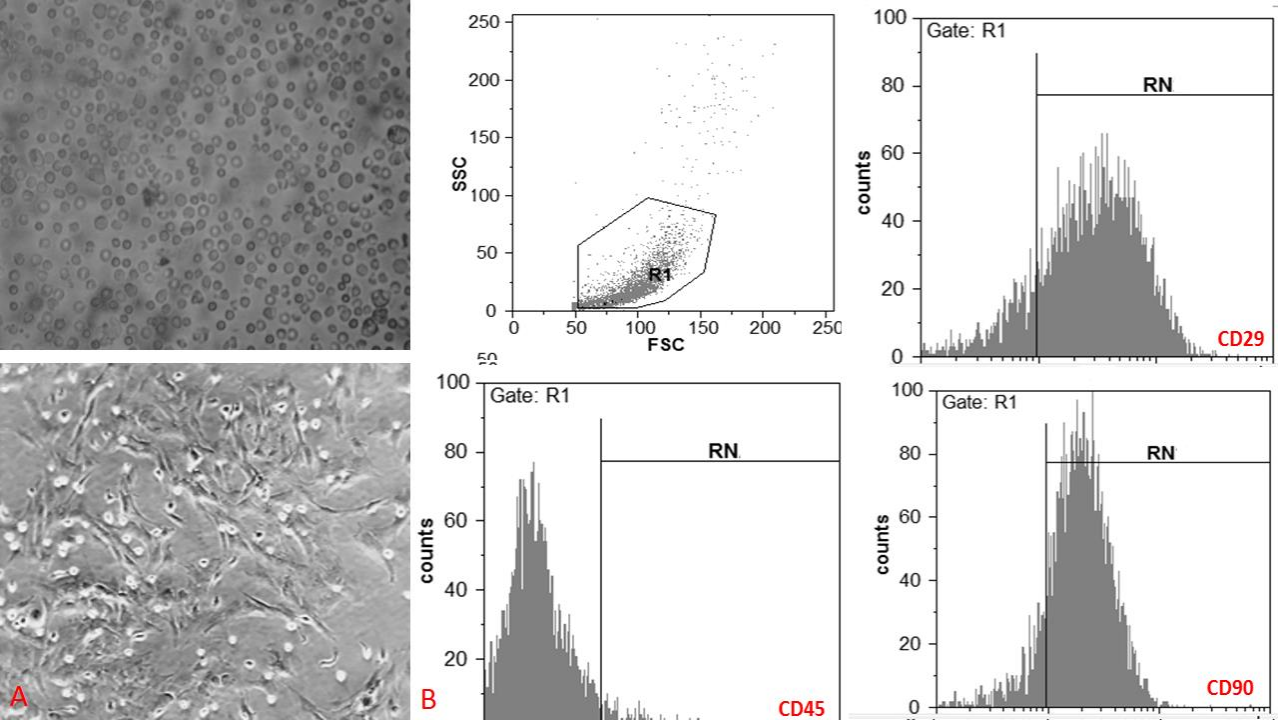
Characterization of mesenchymal stem cells (MSCs).

In the present investigation, we observed that the bone marrow isolated cells gradually appeared as a homogeneous fibroblast-like, spindle-shaped morphology (Figure 1 A). When data were analyzed by flowcytometry method, it has been demonstrated that subculture 3 of the adherent isolated cells (from the bone marrow of rats) have expressed specific marker for MSC (CD29 and CD90); however, the expression of hematopoietic cells marker (CD 45) was not found on their surfaces (Figure 1 B). Therefore, as it is visible, the subculture 3 adherent cells represented a homogeneous fibroblast-like, spindle-shaped morphology, which is a typical feature for the MSCs (Figure 1 B).

**A)** Illustrates a representative field of bone marrow-derived MSCs in different passages. Upper image: At the first, the isolated cells by bone marrow aspiration illustrate a round morphology. Lower image: The subculture 3 of the cells illustrates typical fibroblast-like morphology of MSCs. **B) **Immunophenotype of MSCs**.** The data indicated that the subculture 3 of the adherent isolated cells from the bone marrow of rats showed the MSC marker of rats (CD29 and CD90); nonetheless, they were negative for the marker of the hematopoietic cell (CD 45). Data are shown as the mean ±SD

**Figure 2:**
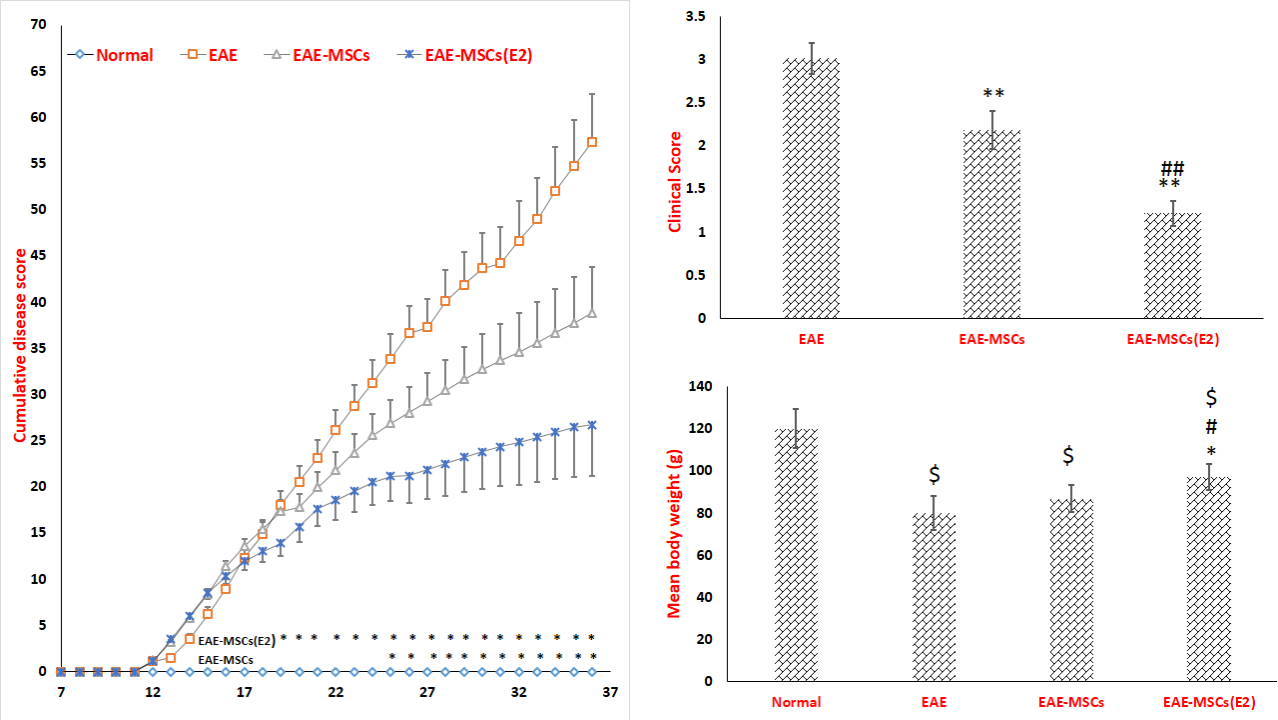
The clinical evaluation of experimental autoimmune encephalomyelitis (EAE)

- Evaluation of cumulative disease disability. **B**) Assessment of the mean clinical score. **C**) The mean body weight of rats during the experiment. Data are reported as the mean ±SD. (*P<0.01, **P<0.001 versus EAE group; # *P*<0.01, ##*P*<0.001 versus EAE rats that received untreated mesenchymal stem cells (MSCs), $ *P*<0.01 versus normal rat).

**Figure 3:**
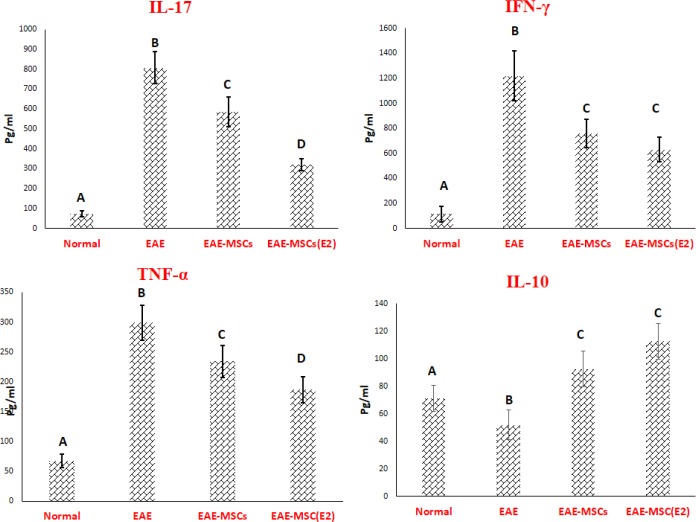
*Ex vivo* evaluation of cytokines production by splenocytes

Data are shown as the mean ±SD. (Different letters represent a significant difference at *P* < 0.01).

**Figure 4:**
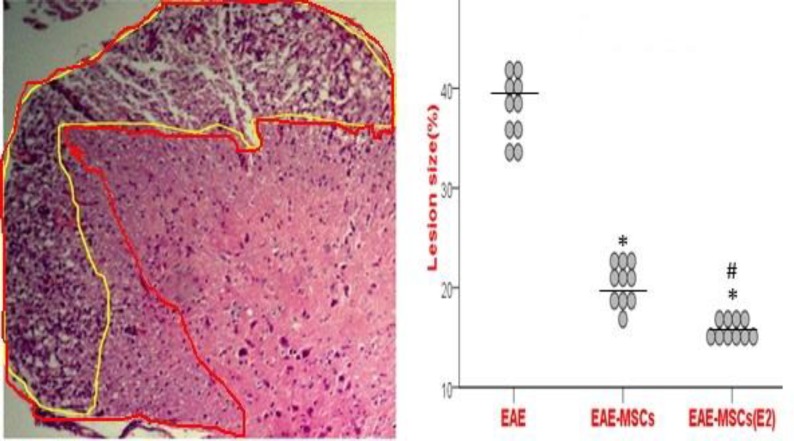
Neuropathological examination of spinal cord sections

The 200x magnification was used for analyzing of the total white matter area as well as the size of white matter lesions. Each point is the value of an individual rat. The line drawn across each experimental autoimmune encephalomyelitis (EAE) group demonstrates the mean. The yellow line shows the damaged area and the red line represents the whole white matter. (*P <0.001 versus EAE group; # *P* <0.001versus EAE rats that received untreated mesenchymal stem cells (MSCs)).

**Figure 5:**
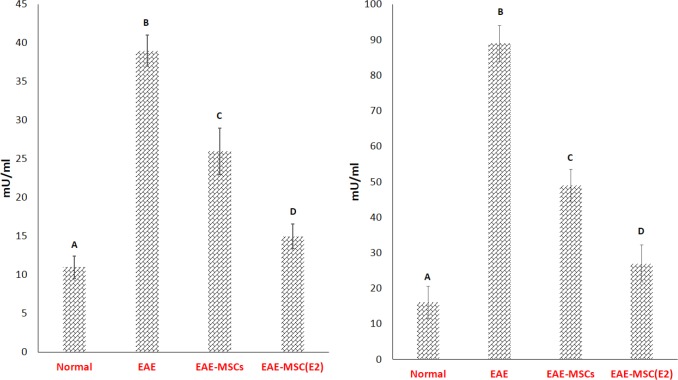
Assessment of the myeloperoxidase activity (A) and nitric oxide (B) in the sera of experimental autoimmune encephalomyelitis (EAE) rats

Results are expressed as the mean ±SD. (Different letters represent a significant difference at *P* < 0.001).

The analysis of disability scores showed that EAE rats that received 17β-ED primed MSCs displayed a remarkable abolishment in the cumulative disease disability compared to EAE rats receiving untreated MSCs (Figure 2 A). The time scale for reducing symptoms was initiated from day 19 post-induction for EAE rats that received 17β-ED primed MSCs; however, as we have observed, this time point was the day 25 post-induction in EAE rats receiving untreated MSCs (Figure 2 A). Findings also revealed that EAE rats had a markedly regression in mean clinical score (Figure 2B). Albeit, the average means clinical score exhibited more considerable difference regression in the EAE animals treated with 17β-ED primed MSCs compared to the EAE rats and those received unprimed MSCs (Figure 2 B). Accordingly, the weight elevation was considerably disrupted in EAE rats, so that the mean body weight in this group was significantly decreased when compared to the normal rats (Figure 2C). However, the only, treatment of EAE rats with 17β-ED primed MSCs significantly restricted the extent of weight loss compared to the vehicle-treated EAE animals (Figure 2 C).

Following *ex vivo* stimulation, the circulating levels of IL-17, TNF-α, and IFN-γ were significantly increased and reversely, the level of IL-10 was decreased in EAE rats in comparison with normal rats (Figure 3). Current experimental data demonstrated that both cell therapy protocols with 17β-ED primed MSCs caused an apparently decrease in the level of the pathogenic pro-inflammatory IL-17, TNF-α, and IFN-γ cytokines, while inversely increased IL-10 as an anti-inflammatory cytokine by splenocyte population of EAE rats when compared to the vehicle-treated EAE group (Figure 3). As is clearly shown in Figure 4, cell therapy with 17β-ED primed MSCs caused a considerable reduction in IL-17 and TNF-α production by splenocyte population more prominent than these productions by splenocyte population of EAE rats that received untreated MSCs (Figure 4). After treatment, a significant change was observed in the level of the pro-inflammatory IFN-γ and anti-inflammatory IL-10 cytokines by the splenocyte population derived from EAE rats. There exists no significant difference between both cell therapy groups regarding *ex vivo* production of IFN-γ and IL-10 as pro- and anti-inflammatory cytokines, respectively (Figure 3). 

Blind analysis indicated that the demyelinating areas were significantly suppressed in EAE rats receiving 17β-ED primed MSCs compared to EAE rats treated with untreated MSCs (Figure 4). 

As shown in Figure 5‌A, both the activity of the MPO along with the NO levels were remarkably elevated in EAE rats compared to normal rats (Figure 5A and 5B). Present findings also indicated that both of therapies significantly reduced the activity of MPO in EAE rat's circulation when compared to the vehicle-treated EAE rats. However, the MPO activity has demonstrated a more remarkable reduction in the sera of EAE rats that received 17β-ED primed MSCs compared to those received untreated MSCs (Figure 5A).

Similarly, the attained data revealed that the circulatory level of NO was significantly reduced in the sera of EAE rats that received both cell therapies compared to vehicle-treated EAE rats (Figure 5B). Although the NO reduction was more notable in the EAE rats treated with 17β-ED primed MSCs than those received untreated MSCs (Figure 5B).

## Discussion

Regenerative and immunomodulatory properties of the MSCs in treatment of EAE models have been previously reported by several investigators (12, 27, 28). On the other hand, it is now well established that the immunoregulatory functions of MScs are highly regulated through several signaling systems (5, 23, 24, 29). Additionally, it has been evidenced that estradiol plays a prominent parts in regulation of various functions of MSCs, including processes of cell proliferation and vascular endothelial growth factor production (30). It has also been reported that the numbers of circulating MSCs are significantly elevated (by almost 15-fold) in rats when they were maintained in hypoxic conditions for almost 3 weeks (31). Interestingly, it has also been indicated that 17β-ED can induce induction of hypoxia-inducible factor 1a (HIF-1a) (32). While MSCs were treated with 17β-ED, their migration to the injured pancreas was facilitated by HIF-1a (13). Consistently, present findings indicated that in EAE rats, 17β-ED primed MSCs displayed a better outcome than 17β-ED non-treated MSCs. So that this caused regression of the cumulative clinical score and neuropathological changes than untreated MSCs. In other words, the EAE rats treated with 17β-ED primed MSCs showed significant weight increase in comparison with the other groups, including MSCs alone.

Oxidative and nitrative substances derived from microglial cells or other infiltrated cell types are implicated as important mediators for demyelination and axonal injury in both MS and EAE clinical states (33). It is clear that in EAE, the mitochondrial nitration-derived components are responsible for preceding the infiltration of inflammatory cell types and further causing mitochondrial membrane loss and therefore apoptotic cell death (34). Also, it is clear that antioxidant strategies could ameliorate EAE (35). MPO is an enzyme found in the myeloid cells cytoplasm and is involved in catalysis of the reactive oxygen substances and thereby participates in the inflammatory processes (36). Several former documents evidenced that mononuclear cells isolated from blood of MS patients are able to produce huge measures of reactive oxygen species or nitrogen-based substances (37, 38). Moreover, elevated serum concentrations of MPO were found in Japanese patients with conventional and optic spinal MS (39). A pervious investigation reported that therapeutic administration of the MPO- inhibitor (*N*-acetyl lysyltyrosylcysteine amide) could significantly decrease the EAE severity (36). This is in agreement with the results of the current study, which exhibited that the serum levels of MPO and NO were significantly attenuated in EAE rats that received 17β-ED primed MSCs more than the EAE rats that received untreated MSCs.

The EAE pathogenesis is principally mediated by CD4^+^ T lymphocytes specific for autoantigens. In these circumstances, the profiles of cytokines that are produced by infiltrating T lymphocytes play a pivotal part in defining the extent of EAE and MS lesions (1). Nowadays, it is also well established that Th17 cells (interleukin-17-producing T-helper cells) are within the main cell types and are involved in disruption of the blood-brain barrier (BBB) and further neuroinflammation both in MS and EAE model (1, 12). Additionally, T-helper cells (Th1 cells) play an undeniable role in development of CNS injuries (possibly by IFN-γ) occurred in MS or EAE following the disruption of BBB by Th17 lymphocytes (40). Another potent inflammatory cytokine that is produced by several cell types, including Th1 like natural killer (NK) cells, neutrophils, mast cells and eosinophils, is TNF-α. TNF-α is also actively involved in the pathogenesis of EAE and MS via initiation of leukocyte migration toward the CNS as well as directing damage to the oligodendrocytes, myelin, and axons (12). The IL-10 that is produced by a broad spectrum of immune cells is paramountly involved in termination of the inflammation and tissue damage, in an inverse fashion to IFN-γ and TNF-α (41, 42). In consistent with our findings, earlier documents reported that MSC therapy in the EAE models was able to inhibit the production of Th17/Th1 cytokines family members (such as IL-17, TNF-α, and IFN-γ) (43). Moreover, novel finding of the current study demonstrated that the splenocyte-derived pro-inflammatory cytokines, including IL-17 and TNF-α were significantly attenuated in EAE rats that received 17β-ED primed MSCs more than EAE rats that received untreated MSCs. Production of IFN-γ and IL-10 were significantly altered in the splenocytes of the EAE rat following MSCs therapy; however, there exists no considerable difference between the both groups receiving cell therapy. Notably, previous investigations reported that the Th17 cells are more pathogenic than Th1 lymphocytes (44), while we showed that IFN-γ to IL-10 and IL-17 to IL-10 were significantly decreased.

Collectively, based on our results it could probably be claimed that a better clinical and histological outcome of EAE may be achieved in future treatment by17β-ED primed MSCs compared to MSCs alone. This might possibly be due to the further immune deviation of pro-inflammatory IL-17 and TNF-α cytokines along with more decrease in the serum NO and MPO levels. There might also exist some other mechanisms, which remain to be elucidated.

## Conclusion

Overall, findings of the present research for the ﬁrst time revealed that the MSCs treated with 17β-ED caused more favorable regression of the signs and induced better outcome compared to untreated MSCs in EAE model. Thus, the present strategy may probably be regarded as an effective approach for improvement of MS signs and symptoms and regardingly be considered for MS therapy.
